# Awareness of Inflammatory Bowel Disease in Bahrain: A Survey-Based Study

**DOI:** 10.7759/cureus.103635

**Published:** 2026-02-15

**Authors:** Hawra Alalwan, Abdulla Yateem, Abdulrahman Saleh, Mahmood Alawainati, Ghadeer Alasfoor, Razan Saleh, Turki AlAmeel

**Affiliations:** 1 Department of Medicine, King Fahad Specialist Hospital, Dammam, SAU; 2 Department of Medicine, King Hamad American Mission Hospital, A'ali, BHR; 3 Department of Medicine, Johns Hopkins Aramco Healthcare, Dhahran, SAU; 4 Department of Medicine, Royal College of Surgeons in Ireland, Manama, BHR; 5 Department of Family Medicine, Primary Healthcare Centers, Manama, BHR; 6 Department of Medicine, Salmaniya Medical Complex, Manama, BHR

**Keywords:** awareness, bahrain, crohn’s disease, inflammatory bowel disease, ulcerative colitis

## Abstract

Background: Limited awareness of inflammatory bowel disease (IBD) can lead to diagnostic delays and increased risk of complications. Although awareness levels may vary across demographic and educational groups, there is a notable paucity of research investigating IBD awareness in Bahrain. To address this gap, the present study aims to evaluate the awareness levels of IBD among the Bahraini population.

Methodology: A cross-sectional survey was conducted at 10 primary healthcare centers in Bahrain using a multistage sampling technique. Adult individuals without a prior history of IBD were invited to complete validated questionnaires. Each correct response received a score of one, with a maximum score of three.

Results: A total of 516 individuals completed the questionnaire. Two hundred seventy-six (53.5%) participants had never heard of Crohn’s disease (CD), and only 130 (25.2%) correctly identified the organs affected. On the other hand, 184 (35.7%) had never heard of ulcerative colitis (UC), whereas only 199 (38.6%) correctly identified the organ involved. The overall combined knowledge score for CD and UC was 1.65 ± 1.78 (mean ± SD), with slightly better results for UC (1.00 ± 1.12) than CD (0.65 ± 0.97). Higher education, healthcare occupation, and family history of IBD were significantly associated with greater IBD knowledge (*P* < 0.001, *P* < 0.001, and *P* < 0.003, respectively).

Conclusions: Findings reveal a significantly low level of IBD awareness among the public in Bahrain. This underscores the need for initiatives aimed at enhancing public knowledge of IBD, which could improve early diagnosis and treatment outcomes for affected individuals.

## Introduction

Inflammatory bowel disease (IBD) is a chronic systemic inflammatory disorder with a predilection to the gastrointestinal tract [1]. It is comprised of Crohn's disease (CD) and ulcerative colitis (UC). Although IBD mainly affects the intestinal tract, it may also involve other organ systems in the body, such as the skin, eyes, and joints [2]. Several factors contribute to the complex pathogenesis of IBD, including host genetics, dysregulated immune responses, intestinal microbiota, and environmental factors [3].

The global burden of IBD is increasing worldwide as the prevalence of IBD has increased over the years in both industrialized and newly industrialized nations, including the Arab world [4-5]. A meta-analysis of 16 articles assessing the clinical epidemiology in seven Arab countries revealed a growing incidence of IBD in the Arab world. The incidence was estimated to be 2.33 and 1.64 per 100,000 persons per year for UC and CD, respectively [6]. In Bahrain, the average number of reported IBD cases has increased from three to 14 cases per year in the period between 1984 and 2014 [7].

The diagnosis of IBD can be difficult. This, in addition to a lack of awareness about the disease, may result in a delay in the diagnosis. Data from the Swiss IBD cohort study has estimated the median diagnostic delay in CD to be nine (3-24) months. The length of diagnostic delay was associated with an increased risk of bowel stenosis, intestinal and perianal fistulas, and need for surgery [8]. In UC, diagnostic delay was estimated to be three (interquartile range (IQR) 2-10) months, with more patients experiencing extraintestinal manifestations (EIM) when the diagnosis was delayed [9].

Previous studies have investigated the awareness of IBD worldwide and have reported an overall low level of awareness among the general population in different community sectors. Young age, female gender, and higher level of education were associated with better awareness [10-12]. To date, there are no published studies assessing the awareness of IBD in the general population in Bahrain, therefore, the purpose of this study was to assess the public awareness of IBD and the variables affecting it, such as level of education and having a family member affected by IBD.

## Materials and methods

Study design and population

This was a cross-sectional study conducted on adults attending primary healthcare centers in Bahrain using a multistage sampling technique. Two primary healthcare centers were selected randomly from each of the five health regions in Bahrain. Patients attending these selected centers were invited to participate in the study. Those with a personal history of IBD were excluded. The study protocol was reviewed and approved by the Research Committee of Primary Health Care in the Kingdom of Bahrain on May 5, 2024 (Approval No. PHCRC/TOR/0012/2024).

Research tool and data collection process

Data were collected using a self-administered online questionnaire through Google Forms. The questionnaire was administered by asking participants to scan a barcode to answer the questions under the direct supervision of a member of the research team.

The research tool was a questionnaire that assessed public awareness of IBD. The questionnaire, which was previously used in an Austrian study, was translated into Arabic according to the World Health Organization (WHO) criteria by a group of researchers in Saudi Arabia [11-12]. It consisted of two parts: the first part of the questionnaire gathered basic demographic data and independent variables, including personal or family history of IBD, a history of experiencing specific symptoms, and any prior endoscopic procedures or surgeries involving the colon or small bowel. These independent variables were used to help identify factors associated with IBD awareness and examine potential correlations with the results obtained from the awareness assessment. The second part of the questionnaire included six questions about general knowledge of UC and CD. Each question was assigned a score of one for the appropriate answer, with the possibility of having more than one appropriate answer in the first question. The maximum score was 3. Moreover, the respondents were asked whether they believed there was adequate awareness of IBD or if there should be more public awareness on the matter (Appendices A-C).

Pilot study

A pilot study was conducted with 18 participants to evaluate the reliability of the questionnaire. Reliability was assessed by calculating Cronbach's alpha coefficient, which was estimated to be 0.739, indicating acceptable internal consistency and demonstrating that the questionnaire was reliable for use in the main study.

Data analysis plan

Data were analyzed using SPSS (version 25.0; IBM Corp, Armonk, NY). Data were presented as frequencies and percentages for categorical variables, and as means and standard deviations for continuous variables. t-test and analysis of variance (ANOVA) were used to compare the means of the continuous variables. For all analyses, *P*-values of less than 0.05 were considered significant.

## Results

From September to October 2024, a total of 569 individuals were asked to complete the questionnaire; 53 of them refused to participate, resulting in a response rate of 90.69%.

Out of the 516 respondents, 286 (55.4%) were male. The average age of the participants was 37.9 years, and 163 (31.6%) were under 30 years old. The majority of the respondents were Bahraini nationals (430, 83.3%). Additionally, 228 participants (44.2%) were employed, 27 (5.2%) of whom were healthcare workers. Regarding educational level, 207 participants (40.8%) completed secondary school and 190 (36.8%) were college graduates, while only 25 (4.8%) were illiterate. Moreover, only 17 respondents (3.3%) had a family history of IBD, and 50 (9.7%) had experienced hematochezia before. Furthermore, 496 participants (96.1%) reported they had never undergone any bowel or colonic surgeries, and 409 (79.3%) had never undergone any endoscopic procedure. Sociodemographic and baseline characteristics of the participants are shown in Table 1.

**Table 1:** Sociodemographic and baseline characteristics of the participants.

Variables		*n* (%)
Sex	Male	286 (55.4)
	Female	230 (44.6)
Age (mean ± SD)		37.99 ± 13.87
Age groups	<30 years	163 (31.6)
	30-39 years	136 (26.4)
	40-49 years	107 (20.7)
	50 and above	110 (21.3)
Nationality	Bahraini	430 (83.3)
	Non-Bahraini	86 (16.7)
Educational level	No formal education	25 (4.8)
	Primary school	14 (2.7)
	Middle school	47 (9.1)
	Secondary school	207 (40.1)
	College level	190 (36.8)
	Post-graduate studies	33 (6.4)
Employment status	Employed	228 (44.2)
	Unemployed	158 (30.6)
	Retired	72 (14)
	Student	58 (11.2)
Are you currently working in the healthcare field?	Yes	27 (5.2)
	No	489 (94.8)
Do you have a family history of inflammatory bowel disease (IBD)?	Yes	17 (3.3)
	No	470 (91.1)
	I do not know	29 (5.6)
Have you ever experienced hematochezia?	Yes	50 (9.7)
	No	429 (83.1)
	I do not know	37 (7.2)
Have you undergone any previous surgeries involving the small intestine or colon?	Yes	15 (2.9)
	No	496 (96.1)
	I do not know	5 (1)
Have you ever undergone an endoscopy (gastroscopy or colonoscopy)?	Yes	103 (20)
	No	409 (79.3)
	I do not know	4 (0.8)

Regarding awareness of the CD, 276 participants (53.5%) had never heard of or read about CD, while only 42 (8.1%) had some information about the disease. Furthermore, only 130 respondents (25.2%) knew that CD affects the intestine, and 421 (81.6%) were unaware that there is medical treatment for CD. Regarding UC, 184 participants (36%) had not heard of or read about it, while 133 (25.8%) might have read or heard about it. In addition, 199 respondents (38.6%) correctly identified that UC affects the large bowel and 382 (74%) were unaware that treatment for UC exists. Results are shown in Table 2. 

**Table 2:** Levels of knowledge about Crohn’s disease and ulcerative colitis among the participants.

Statement		*n* (%)
Have you read about Crohn’s disease (CD)/dealt with a CD patient?	Did not hear or read about CD	276 (53.5)
	I might have heard about CD somewhere	75 (14.5)
	I got some information regarding this disease, and I read about it	42 (8.1)
	Personally, I dealt with this disease, or one of my family members did	16 (3.1)
	I do not know	107 (20.7)
CD can mainly affect:	Head/heart	1 (0.2)
	Liver	2 (0.4)
	It is an infectious disease	15 (2.9)
	Small/Large intestine	130 (25.2)
	I do not know	368 (71.3)
Is there a medical therapy for CD?	Yes	71 (13.8)
	No	24 (4.7)
	I do not know	421 (81.6)
Have you heard, read about Ulcerative colitis (UC), or dealt with a UC disease patient?	Did not hear or read about UC	184 (35.7)
	I might have heard about UC somewhere	133 (25.8)
	I got some information regarding this disease, and I read about it	56 (10.9)
	Personally, I dealt with this disease, or one of my family members did	20 (3.9)
	I do not know	123 (23.8)
UC can mainly affect:	Head/heart	2 (0.4)
	Liver	7 (1.4)
	It is an infectious disease	6 (1.2)
	Large intestine	199 (38.6)
	I do not know	302 (58.5)
Is there a medical therapy for UC?	Yes	108 (20.9)
	No	26 (5)
	I do not know	382 (74)

The respondents’ combined average awareness score for IBD was 1.65 ± 1.78 (mean ± SD), with higher awareness levels toward UC compared to CD (1.00 ± 1.12 and 0.65 ± 0.97, respectively).

When comparing IBD knowledge among different variables, a significant difference was observed across educational levels. Participants holding college and postgraduate degrees scored significantly higher than those with lower educational attainment (2.23 ± 1.84, 3.55 ± 2.2, respectively; *P *< 0.001). Moreover, employees and students demonstrated higher average scores (1.93 ± 1.87, 2 ± 1.69, respectively) compared to unemployed and retired respondents (1.24 ± 1.59, 1.35 ± 1.76, respectively) (*P* < 0.001). Notably, healthcare workers had higher average scores (3.19 ± 2.08) than employees in other fields (*P* < 0.001) and participants with a family history of IBD also had higher scores (2.88 ± 2.26) compared to participants without a family history of IBD (1.57 ± 1.73; *P *< 0.003).

There was no significant difference in awareness levels between males and females, as both had approximately similar scores (1.76 ± 1.82, 1.5 ± 1.73, respectively, *P *= 0.102). This was also noted among Bahraini and non-Bahraini nationals (1.69 ± 1.76, 1.44 ± 1.89, respectively, *P *= 0.242). In addition, there was no significant difference in awareness scores for participants who had experienced hematochezia, undergone intestinal surgeries, or undergone gastroscopy or colonoscopy, compared to those who had not, as shown in Table 3. 

**Table 3:** Association between sociodemographic and baseline characteristics of the participants and their knowledge of IBDs. *t-test. **Analysis of variance. IBD, inflammatory bowel disease

Sociodemographic and baseline characteristics		Combined (mean ± SD)	*P*-value	Test value
Sex	Male	1.76 ± 1.82	0.102*	1.637
	Female	1.5 ± 1.73		
Nationality	Bahraini	1.69 ± 1.76	0.242*	1.172
	Non-Bahraini	1.44 ± 1.89		
Age groups	<30 years	1.99 ± 1.71	0.006**	4.168
	30-39 years	1.68 ± 1.92		
	40-49 years	1.50 ± 1.67		
	50 years and above	1.25 ± 1.75		
Educational level	No formal education	0.12 ± 0.44	<0.001**	27.025
	Primary school	0.43 ± 1.16		
	Middle school	0.51 ± 1		
	Secondary school	1.34 ± 1.44		
	College level	2.23 ± 1.84		
	Post-graduate studies	3.55 ± 2.2		
Employment status	Employed	1.93 ± 1.87	<0.001**	6.350
	Unemployed	1.24 ± 1.59		
	Retired	1.35 ± 1.76		
	Student	2 ± 1.69		
Are you currently working in the healthcare field?	Yes	3.19 ± 2.08	<0.001*	4.702
	No	1.56 ± 1.73		
Do you have a family history of IBD?	Yes	2.88 ± 2.26	<0.003**	5.526
	No	1.57 ± 1.73		
	I do not know	2.1 ± 1.97		
Have you ever experienced hematochezia (the passage of fresh blood through the anus)?	Yes	1.68 ± 1.68	0.976**	0.025
	No	1.65 ± 1.78		
	I do not know	1.59 ± 1.92		
Have you undergone any previous surgeries involving the small intestine or colon?	Yes	1.4 ± 1.64	0.356**	1.034
	No	1.67 ± 1.79		
	I do not know	0.6 ± 0.89		
Have you ever undergone an endoscopy (Gastroscopy or colonoscopy)?	Yes	1.68 ± 1.82	0.756**	0.281
	No	1.65 ± 1.78		
	I do not know	1 ± 1.41		
In your opinion, is there sufficient awareness about IBD?	Yes	1.78 ± 1.94	<0.001**	10.367
	No	1.99 ± 1.85		
	I do not know	1.26 ± 1.59		
Do you believe that there should be awareness campaigns about IBD?	Yes	1.81 ± 1.81	<0.001**	11.346
	No	0.7 ± 1.89		
	I do not know	0.86 ± 1.34		

When asked about the sufficiency of IBD awareness, 246 participants (47.5%) reported that awareness of IBD is insufficient, and 427 (82.9%) believed that there should be more public awareness campaigns for IBD.

## Discussion

This study demonstrated an overall low level of awareness of IBD among the general population in Bahrain. Factors such as working in the healthcare field, a higher level of education, and a family history of IBD were associated with better awareness levels.

A low level of awareness was similarly observed in two studies done in Saudi Arabia. Alqahtani et al. revealed a low level of IBD knowledge among the Saudi general population, and Meeralam et al. concluded that the level of awareness was unacceptable [10-11]. On the other hand, Aldakhil et al. did a study on 648 participants in the Alqassim area; their study found a good level of awareness, with more than half of the participants exhibiting good awareness of various aspects of IBD, like symptoms, risk factors, and complications [13]. Reasons for these differences in awareness might include geographical variations, in addition to the fact that most of the study participants were young and educated.

Gender did not pose a significant difference in awareness in our study (*P *= 0.102). However, in the study by Meeralam et al., females had higher average scores in awareness than males [11]. Additionally, Alkully et al. assessed the adequacy of IBD knowledge in 473 Saudi participants and found that females had a significantly more adequate level of knowledge compared to men [14]. Generally, women engage more in health-seeking behaviours and are more open to communicating about their health than men [15-16].

When comparing knowledge of IBD across different academic levels, there was a significant association between the knowledge level of IBD and respondents' educational level (*P *< 0.001). Specifically, respondents with higher educational achievements had higher awareness compared to their counterparts. This observation was seen in many other studies, and this highlights the importance of targeting the lower educational groups [10-11,13].

As expected, healthcare workers exhibited better awareness compared to non-healthcare workers (*P *< 0.001). This aligns with the study by Aldakhil et al. which looked into the level of awareness among participants who had a professional medical background compared to other professionals and found that the former had significantly higher levels of awareness about IBD [13]. In addition, a study by Eid et al. investigated where participants are getting their information about IBD and found that about half cited healthcare professionals as their primary source, followed by awareness campaigns [17].

Family history of IBD is one of the strongest risk factors for developing IBD [18], and the present study demonstrated that participants who have a family history of IBD scored significantly higher than their counterparts (*P *< 0.003). In the study by Aldakhil et al., most participants were aware that family history of IBD is a risk factor for developing IBD; additionally, these participants exhibited better awareness of IBD [13].

When assessing factors that could be associated with better awareness levels, we found that experiencing symptoms like hematochezia or having any endoscopic procedure or intestinal surgery were not significantly associated with better awareness of IBD.

This study has several strengths. It is the first nationwide study assessing the awareness of IBD in Bahrain, and it was supported by a large sample size that included both nationals and expats. In addition, participants were asked to participate in the questionnaire in person with access to instructions when needed. However, our study had limitations; the participants of the study were only recruited from primary healthcare centers, and the results may not reflect individuals who do not engage with public health services. Larger-scale future studies are needed to assess the generalizability of the results and to further identify factors affecting the awareness of IBD in the remaining patient population.

## Conclusions

Although this study had limitations, our findings suggest that the low level of awareness surrounding IBD continues to challenge patients, physicians, and the healthcare system. Lower educational levels and non-medical professions are associated with even lower levels of awareness. Addressing this gap through education and awareness tailored to certain patient populations is critical to improve the outcome and quality of life for individuals living with this condition.

## Appendices

Appendix A 

**Table 4:** Demographic data questionnaire

Parameters	Count	Percentage
Gender		
Male		
Female		
Age group		
17-19		
20-29		
30-39		
40-49		
50-59		
>60		
Employment status		
Employed		
Unemployed		
Retired		
Student		
Educational level		
Illiterate		
Primary		
Intermediate		
Secondary		
Bachelor		
Masters		
PhD		

Appendix B 

**Table 5:** Crohn's disease questionnaire [11-12]

Responses	Score assigned	Count	Percentage
Did you hear, read, or deal with Crohn’s disease?			
I did not hear or read about it	0		
I heard about it somewhere	1		
I got some information about it	1		
I dealt with somebody with the disease	1		
I do not know	0		
Crohn’s disease can affect			
Head	0		
Liver	0		
Intestine	1		
It is an infectious disease	0		
I do not know	0		
Is there medical therapy for Crohn’s disease?			
Yes	1		
No	0		
I do not know	0		
Total score	3		

Appendix C 

**Table 6:** Ulcerative colitis questionnaire [11-12]

Responses	Score assigned	Count	Percentage
Did you hear, read, or deal with Ulcerative colitis?			
I did not hear or read about it	0		
I heard about it somewhere	1		
I got some information about it	1		
I dealt with somebody with the disease	1		
I do not know	0		
Ulcerative colitis disease can affect			
Head	0		
Liver	0		
Intestine	1		
It is an infectious disease	0		
I do not know	0		
Is there medical therapy for Ulcerative colitis?			
Yes	1		
No	0		
I do not know	0		
Total score	3		
